# Chronic Glymphatic Dysfunction Modulates Domain‐Specific Cognitive Recovery After Stroke: A DTI‐ALPS Lesion Stratification Study

**DOI:** 10.1111/cns.70512

**Published:** 2025-07-14

**Authors:** Qingwen Chen, Tao Zhong, Jian Liu, Binke Yuan, Han Gao

**Affiliations:** ^1^ Department of Neurosurgery The Affiliated Qingyuan Hospital (Qingyuan People's Hospital), Guangzhou Medical University Qingyuan China; ^2^ Department of Neurosurgery The First Affiliated Hospital of Guangdong Pharmaceutical University Guangzhou China; ^3^ Key Laboratory of Brain, Cognition and Education Sciences, Ministry of Education Institute for Brain Research and Rehabilitation, South China Normal University Guangzhou China; ^4^ Philosophy and Social Science Laboratory of Reading and Development in Children and Adolescents (South China Normal University), Ministry of Education Guangzhou China

**Keywords:** chronic stroke, cognitive recovery, DTI‐ALPS, glymphatic system, post‐stroke cognitive impairment

## Abstract

**Background and Aims:**

Glymphatic dysfunction may exacerbate post‐stroke cognitive impairment (PSCI) via impaired metabolic waste clearance. However, longitudinal dynamics of glymphatic function during the chronic stroke phase (3–12 months) and links to cognitive recovery remain unclear. This study aimed to characterize chronic glymphatic remodeling dynamics using the DTI‐ALPS index while exploring its temporal associations with cognitive outcomes and assessing lesion location effects.

**Methods:**

In this retrospective cohort study, 51 chronic stroke patients (3–12 months post‐stroke) and 27 matched healthy controls underwent DTI scans and neuropsychological assessments (evaluating language, memory, motor, attention) at 3 months (3 M‐S) and 1 year (1Y‐S) post‐stroke. The DTI‐ALPS index was calculated for lesioned/contralateral hemispheres. Patients were stratified by lesion location (cortical [*n* = 17] vs. subcortical [*n* = 34]). Group comparisons and Spearman correlations (FDR‐corrected) were performed.

**Results:**

Stroke patients showed significantly lower DTI‐ALPS index versus controls at both 3 M‐S and 1Y‐S (FDR‐*p* < 0.001). At 3 M‐S, the lesioned hemisphere ALPS index was significantly lower than the contralateral hemisphere (FDR‐*p* < 0.05); this difference resolved by 1Y‐S. No significant differences existed between cortical/subcortical subgroups. Weak correlations emerged at 3 M‐S between lesioned‐hemisphere ALPS index and Motor/Memory scores (*r* = 0.280–0.316, uncorrected *p* < 0.05), but these did not survive FDR correction and disappeared by 1Y‐S. Lesion volume did not correlate with ALPS index.

**Conclusions:**

Chronic stroke patients exhibit persistent glymphatic dysfunction. The affected hemisphere showed more severe impairment at 3 months post‐stroke, with partial improvement observed by the 1‐year mark. Transient cognitive associations observed at 3 months diminished by the 1‐year follow‐up, suggesting stabilization of recovery patterns in later stages. Despite study limitations, these findings validate the utility of the DTI‐ALPS index for chronic‐phase assessments and highlight the importance of targeting glymphatic dysfunction as a therapeutic strategy for PSCI.

## Graphical abstract

Chronic stroke patients exhibit persistent glymphatic dysfunction (reduced DTI‐ALPS index), with lesioned hemispheres showing greater impairment at 3 months post‐stroke. Partial functional recovery occurs by 1 year, resolving interhemispheric differences. Transient weak correlations between ALPS index and motor/memory scores at 3 months disappear at 1 year, indicating stabilized recovery patterns. Lesion location and volume show no significant impact. These findings validate DTI‐ALPS for chronic‐phase monitoring and highlight glymphatic restoration as a therapeutic target for PSCI.

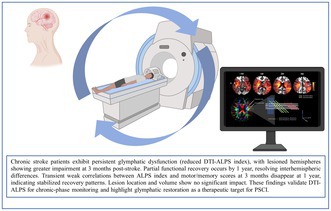

## 1 Introduction

The pathogenesis of post‐stroke cognitive impairment (PSCI) is closely associated with impaired cerebral metabolic waste clearance, where glymphatic system (GS) dysfunction may exacerbate cognitive decline through abnormal β‐amyloid (Aβ) deposition and neuroinflammatory cascades [1, 2]. Animal studies demonstrate that ischemic injury disrupts the polarized distribution of aquaporin‐4 (AQP4), thereby suppressing cerebrospinal fluid–interstitial fluid exchange [3]. Clinical neuroimaging evidence further reveals that acute stroke patients exhibit compromised glymphatic function, correlating with enlarged white matter hyperintensity volumes and early cognitive decline [4]. However, systematic investigations into the dynamic remodeling of glymphatic function during the chronic stroke phase (3–12 months post‐stroke) and its association with long‐term cognitive outcomes remain scarce.

In stroke patients, chronic glymphatic dysfunction leads to persistent accumulation of metabolic waste, disrupting neural function and cognitive behavior through a vicious cycle of impaired clearance efficiency and imbalanced neurorepair. Although cerebral perfusion may partially recover during the chronic phase, sustained hypoperfusion damages perivascular spaces and astrocytic AQP4 polarization, chronically restricting glymphatic drainage and promoting pathological deposition of Aβ, tau proteins, and ischemia‐related neurotoxins [5, 6]. This accumulation persistently activates microglia, exacerbates neuroinflammation, and disrupts synaptic plasticity and neural network stability. Prolonged drainage impairment further induces mechanical stress on perivascular basement membranes, exacerbating cerebral small vessel disease (CSVD)‐related pathologies (e.g., white matter hyperintensities, microbleeds), creating a bidirectional glymphatic–CSVD pathological loop that perpetuates subcortical cognitive deficits in executive function and attention [7, 8]. Developing glymphatic function assessment methods could enable early diagnosis, therapeutic evaluation, and rehabilitation prediction.

Diffusion tensor imaging along the perivascular space (DTI‐ALPS) index, quantifying diffusion anisotropy near peri‐ventricular perivascular spaces, has emerged as a non‐invasive biomarker for glymphatic activity [9]. Initial studies demonstrated reduced ALPS index in Alzheimer's disease patients inversely correlating with Aβ burden. Subsequent applications in cerebral small vessel disease [10], traumatic brain injury [11], and acute stroke cohorts (< 3 months) [5] have confirmed ALPS index correlations with neurological outcomes. Nevertheless, extant research predominantly focuses on acute/subacute phases or mixed lesion types, lacking longitudinal assessment of glymphatic functional recovery during chronic stroke stages or anatomical stratification by lesion laterality and cortical–subcortical topography.

Critical methodological limitations persist in previous studies: (1) short‐term or cross‐sectional designs fail to capture temporal dynamics between glymphatic remodeling and cognitive recovery; (2) overlooking endogenous glymphatic repair mechanisms and neuroplastic interactions during chronic phases. These gaps hinder a comprehensive understanding of glymphatic contributions to PSCI. This retrospective cohort study enrolled stroke survivors 3–12 months post‐onset and systematically investigated the trajectory of DTI‐ALPS index during the chronic phase and its correlation with multidimensional cognitive assessments. The study aims to: (1) elucidate chronic glymphatic remodeling dynamics and temporal associations with cognitive recovery; (2) reveal anatomical lesion characteristics as moderators of glymphatic‐cognitive relationships; (3) provide theoretical foundations for personalized glymphatic‐targeted rehabilitation strategies. By implementing refined anatomical stratification and extended observational windows, this study addresses critical knowledge gaps regarding chronic‐phase glymphatic evolution and lesion‐specific effects, offering crucial neuroimaging evidence for optimizing spatiotemporal windows in cognitive rehabilitation.

## 2 Materials and Methods

### 2.1 Participants

**FIGURE 1:**
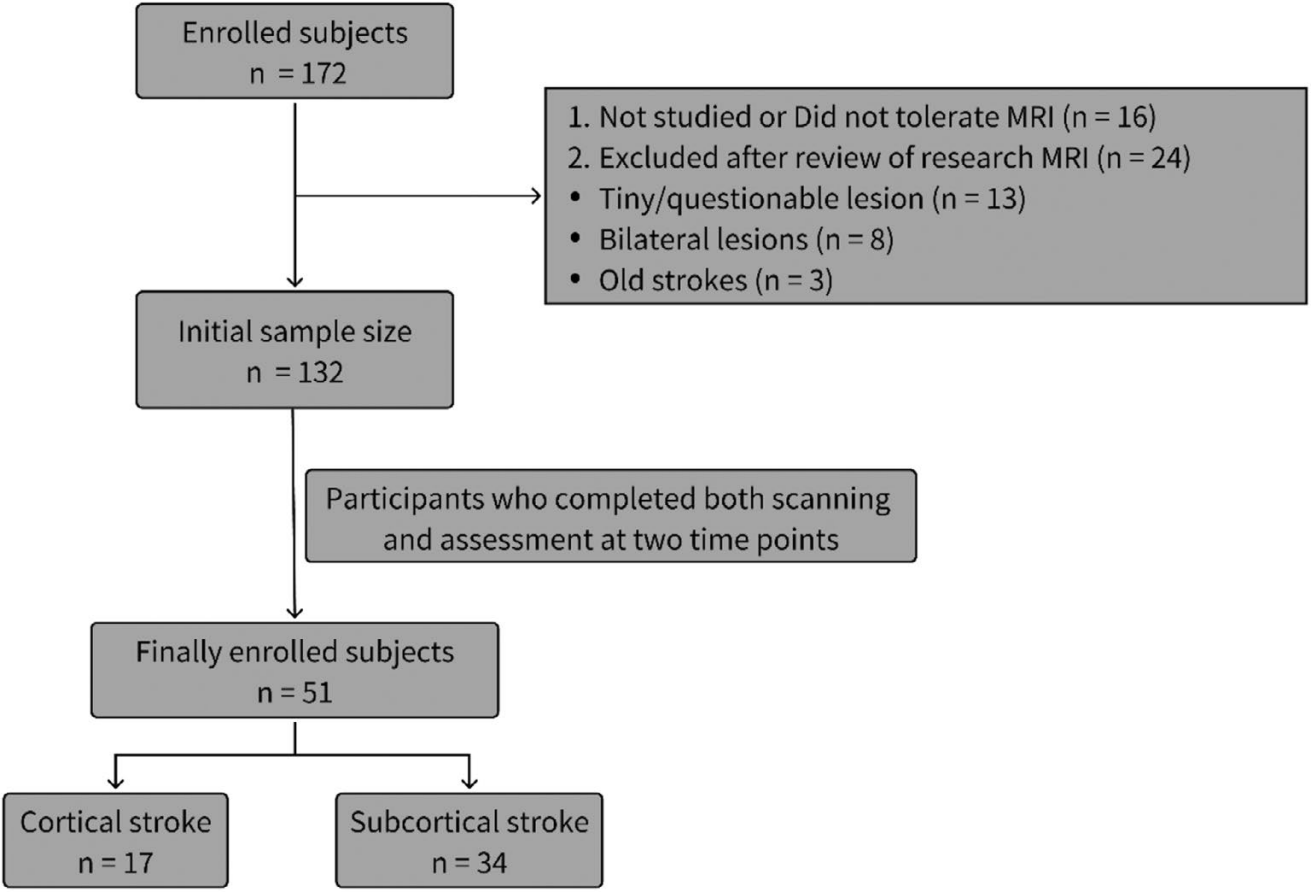
Participant enrollment flowchart for this study.

The stroke dataset we used is part of the Washington Stroke Cohort, which is publicly available at https://cnda.wustl.edu/data/projects/CCIR_00299. Subjects (*n* = 172) were prospectively recruited, of whom 132 met the post‐enrollment criteria. The inclusion and exclusion criteria were detailed in Corbetta et al. [12]. Among the enrolled subjects, lesions were manually segmented using Analyze software (www.mayo.edu) by examining structural images (T1‐weighted, T2‐weighted, and FLAIR) displayed in atlas space. Two neurologists (Maurizio Corbetta and Alexandre Carter) reviewed all segmentations, focusing on distinguishing lesions from cerebrospinal fluid (CSF) and hemorrhage from surrounding vasogenic edema [13]. Fifty‐one stroke patients were included in the analysis, and all underwent imaging scans and neuropsychological testing at both time points. Individual lesion distributions are shown in Figure S1. We divided the 51 patients into 2 groups: (a) based on lesion location, ALPS index were calculated for each patient's lesioned hemisphere and the contralateral hemisphere; (b) cortical stroke and subcortical stroke. For the patients included in the analysis, follow‐up measurements were conducted at 3 months and 1 year post‐stroke. A healthy control group (*n* = 27) was matched to the study sample for age, gender, and years of education. The enrollment process for subjects is illustrated in Figure 1. Written informed consent was obtained from all participants in accordance with the Declaration of Helsinki and procedures established by the Washington University in Saint Louis Institutional Review Board. All aspects of this study were approved by the Washington University School of Medicine (WUSM) Internal Review Board. This study is a retrospective study compliant with the STROBE guideline [14].

### 2.2 Neuropsychological Assessment and Analysis

Subjects were tested at approximately 3 and 12 months post‐stroke, with data also collected from healthy controls. A comprehensive battery of 44 behavioral tests across four domains—language, memory, motor, and attention—was administered to capture common deficits in stroke patients. Principal component analysis (PCA) was used to summarize behavioral measurements, extracting components for each domain: one for language (76.5% variance), two for motor (77.2% variance), two for memory (66.2% variance), and three for attention (57.1% variance) [12]. NHISS scores were assessed at each time point to evaluate recovery effects.

### 2.3 MRI Acquisition

All imaging was performed using a Siemens 3T Tim‐Trio scanner at the Washington University School of Medicine (WUSM) with a standard 12‐channel head coil. The MRI protocol included structural and diffusion tensor scans. Structural scans consisted of: A sagittal MP‐RAGE T1‐weighted image (TR = 1950 ms, TE = 2.26 ms, flip angle = 9°, voxel size = 1.0 × 1.0 × 1.0 mm, slice thickness = 1.0 mm); A transverse turbo spin‐echo T2‐weighted image (TR = 2500 ms, TE = 435 ms, voxel size = 1.0 × 1.0 × 1.0 mm, slice thickness = 1.0 mm); A sagittal FLAIR (fluid‐attenuated inversion recovery) image (TR = 7500 ms, TE = 326 ms, voxel size = 1.5 × 1.5 × 1.5 mm, slice thickness = 1.5 mm). Diffusion tensor scans included multi‐directional and multi‐weighted diffusion‐weighted images (64 directions, b‐value = 1000 s/mm^2^, and one b0 reference image) with the following parameters: TR = 9200 ms, TE = 90 ms, 2.0 mm isotropic voxels, matrix size = 128 × 128, requiring a total acquisition time of 14 min.

### 2.4 DTI Preprocessing and Diffusion Tensor Image Analysis Along the Perivascular Space (DTI‐ALPS)

**FIGURE 2:**
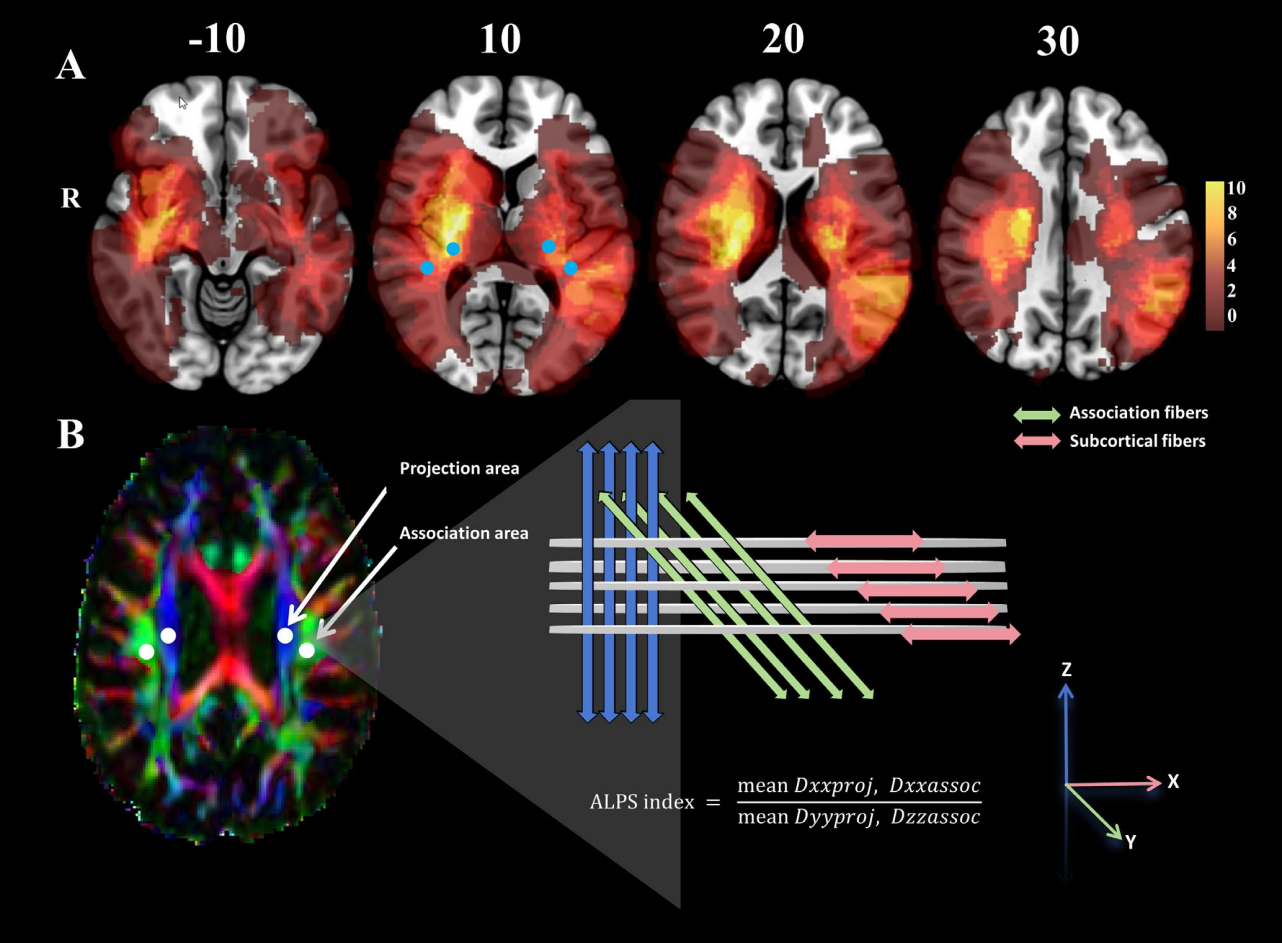
(A) Lesion overlap map of the included stroke patients, with ROIs strategically placed in regions exhibiting minimal lesion overlap. (B) Calculation method and schematic diagram of the DTI‐ALPS index.

DWI data pre‐processing was performed using MRtrix3 (https://www.mrtrix.org). Raw DWI data were converted to MRtrix's “.mif” format, followed by denoising, Gibbs ringing artifact removal, and correction for eddy‐current distortions and head motion using FSL's eddy and topup tools. Bias field correction was applied using the ANTs algorithm. Preprocessed data were converted back to NIfTI format, and tensor‐based diffusion metrics, including fractional anisotropy (FA), mean diffusivity (MD), axial diffusivity (AD), and radial diffusivity (RD), were computed using FSL's “dtifit” command. RD was derived by averaging the second and third eigenvalues (L2 and L3). The FA map was registered to the ICBM FA template, and color‐coded vector maps were generated to encode directional information. The reoriented colored FA map was loaded into the ITK‐SNAP toolbox for manual delineation of regions of interest (ROIs). Circle ROIs of 5‐mm diameter were drawn on the FA map, and fiber orientations and diffusivities along the x‐, y‐, and z‐axes were measured at the voxel level within the ROIs (Figure 2). For each fiber type (projection, association, and subcortical fibers), one voxel with maximum orientation was selected. Dx values in projection (Dxproj) and association (Dxassoc) fibers, Dy values in both fiber types (Dyproj, Dyassoc), and Dz values in both fiber types (Dzproj, Dzassoc) were automatically measured to calculate the ALPS index using the formula:

$$\text{ALPS index}=\;\frac{\text{mean}\;\text{Dxxproj},\;\text{Dxxassoc}}{\text{mean}\;\text{Dyyproj},\;\text{Dzzassoc}}$$

### 2.5 Statistical Analysis

Statistical analyses were conducted using SPSS version 27.0 (IBM Corporation, USA) and R version 4.4.2. Continuous variables were summarized as mean ± standard deviation (SD) or median [interquartile range (IQR)], while categorical variables were expressed as frequencies [percentages (%)]. The Shapiro–Wilk (S–W) test was applied to evaluate the normality of data distribution. For comparisons across the three groups (Control, 3 months post‐stroke [3 M‐S], and 1 year post‐stroke [1Y‐S]), one‐way ANOVA was used for normally distributed variables, while the Mann–Whitney *U* test or Kruskal–Wallis test was employed for non‐normally distributed variables. Post hoc analyses with false discovery rate (FDR) correction were performed to identify specific group differences when significant results were observed. Categorical variables were compared using the chi‐square test or Fisher's exact test, as appropriate. Pearson's correlation was utilized to assess linear relationships between continuous variables, while Spearman's correlation was applied for rank‐based variables. Between‐group comparisons of continuous variables were conducted using independent samples *t*‐tests or Wilcoxon rank‐sum tests, depending on data distribution. Within the stroke group, Spearman correlation was used to evaluate the relationship between the ALPS index and cognitive scores, whereas Pearson correlation was applied to assess the association between infarct volume and the ALPS index. A significance threshold of *p* < 0.05 was applied, with FDR correction for multiple comparisons.

## 3 Results

### 3.1 Demographics and Clinical Information

In this study, stroke patients were stratified into groups based on lesion location. The first group comprised total stroke patients (lesioned hemisphere and contralateral hemisphere; *n* = 51, 23 females and 28 males, age range: 22–77 years, mean age: 53.25 ± 10.56 years). The second group included cortical stroke patients (Cor‐lesion) assessed (*n* = 17, 9 females and 8 males, age range: 22–70 years, mean age: 53.35 ± 12.37 years). Subcortical stroke patients (Sub‐lesion) were evaluated (*n* = 34, 14 females and 20 males, age range: 30–77 years, mean age: 53.21 ± 9.73 years). Additionally, 27 healthy controls (15 females and 12 males, age range: 21–83 years, mean age: 55.30 ± 12.77 years), matched for gender, age, and years of education, were recruited. Demographic and clinical characteristics—including age, gender, years of education, smoking history, and diabetes history—showed no significant differences across groups (*p* > 0.05). However, hypertension prevalence was significantly higher in stroke groups compared to controls (*p* < 0.001). Lesion type distribution did not differ significantly among stroke subgroups (*p* > 0.05), while lesion volume differed significantly between cortical and subcortical patients (*p* = 0.007). Participant characteristics are summarized in Table 1.

**TABLE 1:** Demographics and clinical characteristics.

Characteristics	Controls (*n* = 27)	Stroke (*n* = 51)	*p* value	Cor‐lesion (*n* = 17)	Sub‐lesion (*n* = 34)	*p* value
Age (years), mean ± SD	55.30 ± 12.77	53.25 ± 10.56	0.527	53.35 ± 12.37	53.21 ± 9.73	0.755
Female, *n* (%)	15 (55.6%)	23 (45.1%)	0.379	9 (52.9%)	14 (41.2%)	0.496
Years of education (years)^b^	12 [12; 15]	13 [12; 16]	0.589	14[12; 16]	12.5 [12; 14.5]	0.833
History of smoking, *n* (%)	12 (44.4%)	23 (45.1%)	0.956	5 (29.4%)	18 (52.9%)	0.281
Hypertension, *n* (%)	0 (0%)	34 (66.7%)	**< 0.001** *	13 (76.5%)	21 (61.8%)	**< 0.001** *
Diabetes, *n* (%)	3 (11.1%)	13 (25.5%)	0.135	4 (23.5%)	9 (26.5%)	0.317
Lesion type, *n* (%)^a^						
Ischemic	—	37 (72.5%)		10 (58.8%)	27 (79.4%)	0.062
Hemorrhagic	**—**	9 (17.6%)		3 (17.6%)	6 (17.6%)	
Other	**—**	5 (9.8%)		4 (23.5%)	1 (2.9%)	
Lesion volume (cm^3^)^b^	**—**	7.99 [2.58; 41.28]		32.16 [3.22; 82.92]	5.57 [1.29; 22.25]	**0.007** *

*Note:* Age was analyzed using an independent samples *t*‐test or one‐way ANOVA, while other variables were analyzed using the chi‐square test. Significance of bold values indicates statistical significance of *p*‐values when *p* < 0.05 for improved visual clarity.

* *p* < 0.05.

^a^ Fisher's Exact Test.

^b^ Mann–Whitney *U* test or Kruskal–Wallis test.

### 3.2 DTI‐ALPS Index

**FIGURE 3:**
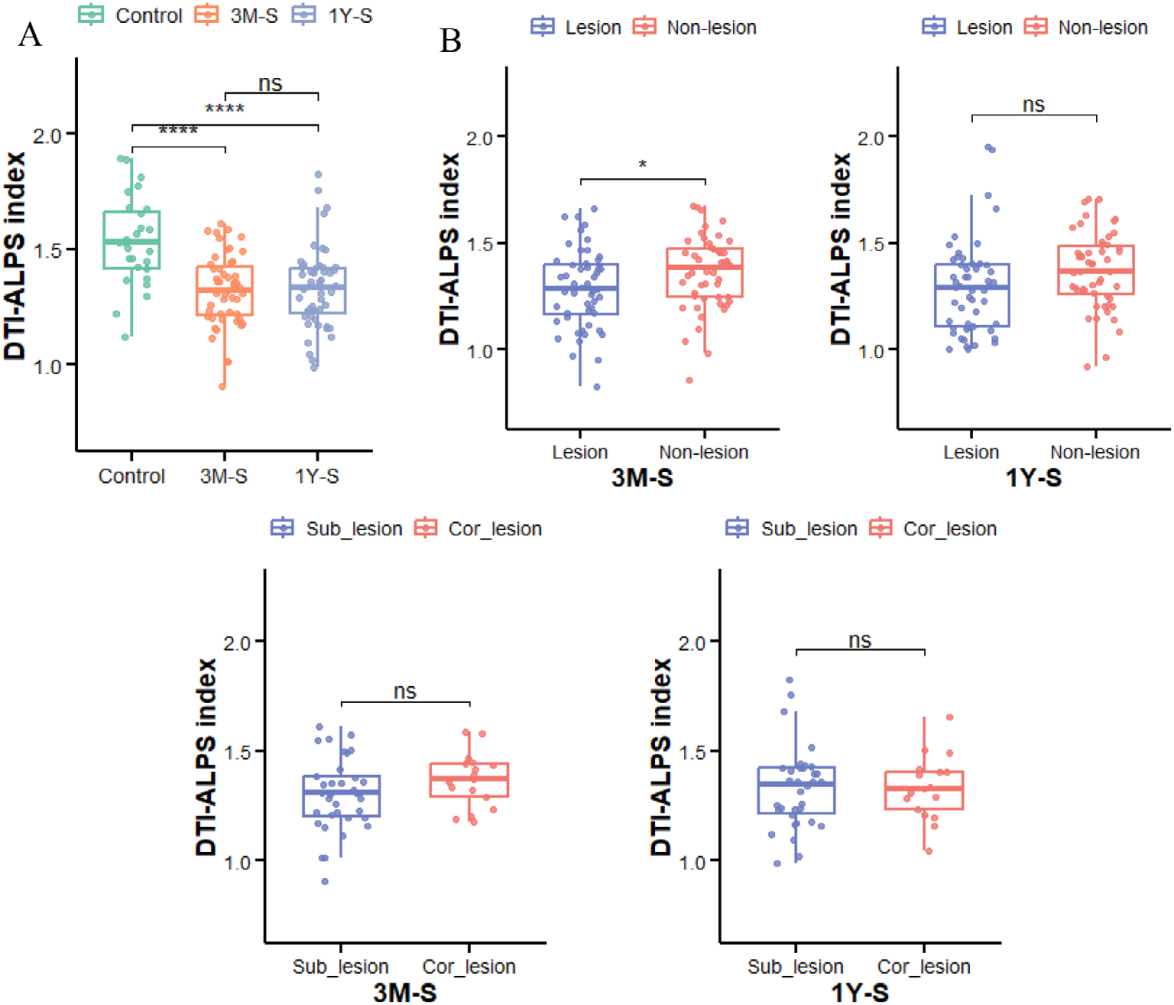
(A) Box plots demonstrating significantly lower DTI‐ALPS index in stroke patients versus healthy controls at both 3 months (3 M‐S) and 1 year (1Y‐S) post‐stroke (FDR‐*p* < 0.001). (B) The plots demonstrate significantly reduced DTI‐ALPS index in lesioned versus contralateral hemispheres at 3 M‐S (FDR‐*p* < 0.05), with this difference becoming non‐significant at 1Y‐S (FDR‐*p* > 0.05). No significant differences in DTI‐ALPS index between cortical (Cor‐lesion) and subcortical (Sub‐lesion) stroke groups at either time point (FDR‐*p* > 0.05). ****p < 0.001, *p < 0.05, ns: not statistically significant.

In this study, we compared the DTI‐ALPS index across different groups. The results revealed significantly lower DTI‐ALPS index in stroke patients versus controls at both time points (FDR‐*p* < 0.001), though no significant changes occurred during the recovery period (FDR‐*p* > 0.05; Figure 3A). At 3 M‐S, the lesioned hemisphere showed a significantly lower DTI‐ALPS index than the contralateral hemisphere (FDR‐*p* < 0.05; Figure 3B), but this difference disappeared by 1Y‐S (FDR‐*p* > 0.05). Similarly, no significant differences were observed between Sub‐lesion and Cor‐lesion groups at either time point (FDR‐*p* > 0.05).

### 3.3 Correlation Between the DTI‐ALPS Index and Cognition

Spearman analysis revealed that only at the 3 M‐S time point, the DTI‐ALPS index in the lesioned hemisphere showed significant correlations with Motor (left/right) scores and MemoryS (*r* = 0.316, 0.300, 0.280; uncorrected *p* = 0.026, 0.034, 0.049, respectively). However, these correlations did not survive FDR correction, and the uncorrected *p* values were of marginal significance. At other time points, no significant correlations were observed between the ALPS index and other cognitive scores (*p* > 0.05; Tables S1–S3).

## 4 Discussion

This study provides a comprehensive investigation into the functional alterations of the glymphatic system (GS) in stroke patients during the chronic rehabilitation phase, with a particular focus on its association with clinical cognitive outcomes. The GS, a critical pathway for brain metabolic waste clearance, plays a pivotal role in maintaining central nervous system homeostasis and supporting higher cognitive functions such as learning and memory [15]. While previous studies predominantly employed invasive techniques like fluorescence tracing or intrathecal contrast agent injection to assess glymphatic function [16, 17], this study leverages the non‐invasive DTI‐ALPS method to indirectly evaluate GS hydrodynamics. Although the clinical applicability of DTI‐ALPS remains debated, accumulating evidence demonstrates its strong correlation with traditional tracer‐based dynamic contrast‐enhanced MRI, as well as its high intraclass correlation coefficients (ICC) and test–retest reliability in neurological disorders such as cerebral small vessel disease and Alzheimer's disease [18, 19, 20]. Our findings further validate the utility of DTI‐ALPS in the stroke rehabilitation phase and underscore the potential link between glymphatic dysfunction and cognitive recovery.

Previous research reveals that stroke patients frequently exhibit glymphatic dysfunction during the acute phase (within 2 weeks post‐onset). Cai et al. [21] reported a significantly lower ALPS index on the lesioned side in patients with acute spontaneous intracerebral hemorrhage (sICH) compared to healthy controls (1.34 ± 0.24 vs. 1.46 ± 0.22, *p* = 0.003). Our study extends these observations to the chronic rehabilitation phase, demonstrating a persistently reduced DTI‐ALPS index in stroke patients relative to controls (FDR‐*p* < 0.001), suggesting delayed functional recovery of the GS. Some studies indicate a gradual increase in the ALPS index 2–3 weeks post‐stroke, with recovery plateauing within 2 months after onset [21, 22]. This non‐linear recovery trajectory may reflect the limited efficacy of conventional interventions, such as anti‐edema therapy or neuroprotective agents, in restoring glymphatic function. Acute neuroinflammation has been implicated in sustained glymphatic impairment through multiple mechanisms [23], including (1) degradation of perivascular basement membranes by pro‐inflammatory cytokines (e.g., IL‐1β, TNF‐α), disrupting CSF‐ISF convective flow; (2) loss of AQP‐4 polarity in astrocytes under neuroinflammatory conditions, reducing directional CSF‐ISF exchange; and (3) accumulation of metabolic waste (e.g., β‐amyloid) in perivascular spaces (PVS) due to reactive microglia‐derived oxidative stress products (e.g., ROS). Furthermore, chronic neuroinflammatory microenvironments exacerbate PVS structural dilation, blood–brain barrier hyperpermeability, and neurovascular uncoupling. While M1 microglia perpetuate pro‐inflammatory cytokine release (e.g., IL‐6), the precise role of TREM2 signaling in AQP‐4 polarization remains unclear [24]. Emerging therapeutic strategies, such as modulating sleep–wake cycles to enhance glymphatic clearance [25] and utilizing non‐invasive techniques like ultrasound to optimize arterial pulsation for CSF‐ISF dynamics, are under investigation, though mechanistic and parametric validation is ongoing.

Based on our study categorizing stroke patients into two groups according to lesion characteristics, Group 1 analyzed lesion‐side ALPS index in 51 patients. Comparative analysis revealed a significantly lower ALPS index in the lesioned hemisphere versus the contralateral hemisphere at 3 months post‐stroke (3 M‐S, *p* < 0.05), with this difference resolving by 1 year post‐stroke (1Y‐S). The ALPS index reduction at 3 M‐S primarily originated from acute‐phase loss of AQP4 water channel polarity in astrocytes, disrupting directional interstitial fluid–CSF flow within PVS and impeding metabolic waste clearance—a process requiring extended recovery time [6]. The resolution of this interhemispheric disparity at 1Y‐S reflects chronic‐phase neurovascular remodeling, including reactive astrocyte end‐foot reconstruction restoring PVS patency, enhanced compensatory drainage from the contralateral hemisphere, and activation of meningeal lymphatic diversion mechanisms, collectively driving bilateral glymphatic functional rebalancing [26]. The second group, stratified by lesion anatomical location (cortical [Cor‐lesion] vs. subcortical [Sub‐lesion]), showed no significant differences in the DTI‐ALPS index between subgroups (FDR‐*p* > 0.05). This may reflect a convergence of pathological impacts on the GS, wherein both cortical infarction‐induced neurovascular coupling disruptions and subcortical lesion‐induced deep venous drainage impairments are mitigated through paraventricular compensatory pathways [3]. This contrasts sharply with findings in brain tumor studies, which demonstrate heterogeneous effects of space‐occupying lesions on the DTI‐ALPS index depending on anatomical location [27]. Collectively, these results suggest that post‐stroke GS functional changes may exhibit non‐lateral dependency and anatomical generalization, with compensatory mechanisms potentially involving (1) dynamic reconstruction of CSF‐ISF exchange pathways, (2) adaptive modulation of neuroglial cell polarization states [28], and (3) coupling between cerebral blood flow autoregulation and glymphatic drainage [29]. The consistency of these findings across two distinct time points further underscores their reliability.

In the dynamic process of neurological recovery after stroke, the functional evolution of the glymphatic system exhibits stage‐specific characteristics. At 3 months post‐stroke, a weak correlation (uncorrected *p* < 0.05) is observed between the DTI‐ALPS index in the lesioned hemisphere and motor/memory scores, which, although not surviving FDR correction, may reflect compensatory activation of the glymphatic system triggered by the resolution of neuroinflammation and peri‐lesional edema absorption [30]. During this phase, edema absorption enhances interstitial fluid flow around motor/memory networks, temporarily improving metabolic waste clearance efficiency, consistent with the gradual recovery of the ALPS index over time after stroke [5]. However, the small effect size (*r* < 0.32) and statistical fragility suggest this signal may be confounded by lesion heterogeneity (e.g., anatomical variations at the cortical–subcortical junction) or comorbid factors (e.g., hypertension‐related perivascular space dysfunction). By 1Y‐S, the disappearance of this correlation signifies glymphatic functional failure, with mechanisms involving persistent loss of AQP4 polarity and perivascular fibrosis [6]. As a key water channel protein in astrocytes, the disrupted circadian rhythm of AQP4 directly impairs interstitial fluid‐cerebrospinal fluid exchange efficiency, decoupling waste clearance from neural repair [31]. Notably, contralateral hemispheric compensation may divert interstitial fluid through alternative meningeal lymphatic pathways, paradoxically exacerbating glymphatic functional decline in the primary lesion area. Additionally, the absence of a significant correlation between lesion volume and the DTI‐ALPS index diverges from previous findings [21], potentially due to the DTI‐ALPS index reflecting local PVS diffusion characteristics rather than the macroscopic space‐occupying effects of overall lesion volume, the greater impact of lesion involvement in key nodes (e.g., basal ganglia, cingulate cortex) on glymphatic function compared to cumulative lesion volume, and the heterogeneity of lesion locations diminishing the significance of volume effects—for instance, small subcortical lesions affecting critical white matter pathways (e.g., posterior limb of the internal capsule or thalamic radiation) may significantly impair glymphatic drainage, whereas larger cortical lesions sparing PVS‐dense regions may minimally alter the DTI‐ALPS index.

Despite these insights, several methodological limitations warrant consideration. First, the absence of acute phase (< 2 weeks) DTI data precludes the capture of dynamic glymphatic dysfunction evolution post‐stroke, particularly during the critical window for neurovascular unit remodeling. Although the single‐center design ensures scanning parameter consistency, the lack of longitudinal acute phase data may obscure the stage‐specific effects of perilesional edema and neuroinflammation on glymphatic drainage, which exhibit time‐dependent regulatory characteristics in animal models. Second, based on our anatomical subgroup analysis (cortical vs. subcortical stroke) employing a binary classification criterion (> 50% cortical lesion volume), this operational but artificial threshold dichotomizes lesions existing along a cortical–subcortical continuum (e.g., basal ganglia‐corona radiata junctional lesions), introducing statistical noise from intragroup heterogeneity. Compounded by the limited sample size in the cortical subgroup (*n* = 17), statistical power is substantially diminished under multiple comparison corrections (e.g., FDR). Consequently: (1) genuine between‐group glymphatic functional differences (such as subtle DTI‐ALPS changes at 1Y‐S) may be obscured; (2) Weak correlation signals (e.g., uncorrected *p* < 0.05 results failing FDR adjustment) become susceptible to classification bias, thus biological interpretations of such marginal findings must be inferred with caution as potential rather than definitive mechanisms. Third, manual region of interest (ROI) delineation may introduce spatial localization bias, underestimating the microstructural heterogeneity of anisotropic diffusion in PVS. Although standardized anatomical landmarks and exclusion of visible vascular abnormalities mitigate this, fully automated segmentation algorithms integrating multimodal imaging features (e.g., FLAIR vascular hyperintensity localization) are needed to enhance the reproducibility and spatial accuracy of DTI‐ALPS measurements.

## 5 Conclusion

This retrospective study analyzed the relationship between DTI‐ALPS index and cognitive behavior in stroke patients during chronic rehabilitation (3–12 months), revealing significantly lower DTI‐ALPS index compared to healthy controls, potentially linked to chronic glymphatic dysfunction. The affected hemisphere showed lower ALPS index than the contralateral side at 3 months, but this lateralization disappeared by 1 year. Weak correlations between ipsilateral ALPS index and motor/memory scores at 3 months diminished over time, reflecting transient glymphatic clearance impacts from early post‐stroke edema resolution, inflammatory responses, or acute‐phase repair processes. The dynamic changes may also involve later neural remodeling, glial reactivity modifications, or compensatory drainage activation (e.g., contralateral/meningeal lymphatic enhancement). Despite limitations including absent acute‐phase imaging and small cortical subgroup samples, this study provides theoretical foundations for glymphatic‐targeted personalized rehabilitation strategies and highlights the importance of chronic‐phase neuroprotective interventions.

## Author Contributions

Qingwen Chen: data curation, conceptualization, formal analysis, methodology, software, writing – original draft, writing – review and editing. Tao Zhong: writing – original draft. Jian Liu: writing – review and editing. Binke Yuan: conceptualization, methodology, formal analysis, writing – review and editing. Han Gao: project administration, resources, writing – review and editing.

## Consent

The authors have nothing to report.

## Conflicts of Interest

The authors declare no conflicts of interest.

## Supporting information


Data S1.

Data S2.

## Data Availability

The stroke dataset has been publicly available at https://cnda.wustl.edu/data/projects/CCIR_00299.
