# Supplementary material for: Chronic Glymphatic Dysfunction Modulates Domain‐Specific Cognitive Recovery After Stroke: A DTI‐ALPS Lesion Stratification Study
Source: CNS Neurosci Ther. 2025 Jul 14;31(7):e70512. doi: 10.1111/cns.70512 (PMC12256992; doi:10.1111/cns.70512)
Supplement: Supplementary file 1 — Data S1. [file CNS-31-e70512-s002.docx]

**Chronic Glymphatic Dysfunction Modulates Domain-Specific Cognitive Recovery After Stroke: A DTI-ALPS Lesion Stratification Study**

Qingwen Chen^1,2^, Tao Zhong^2^, Jian Liu^1^, Binke Yuan^3,4*^, Han Gao^1*^

^1^ Department of Neurosurgery, The Affiliated Qingyuan Hospital (Qingyuan People’s Hospital), Guangzhou Medical University, Qingyuan, China

^2^ Department of Neurosurgery, the First Affiliated Hospital of Guangdong Pharmaceutical University, Guangzhou, China

^3^ Key Laboratory of Brain, Cognition and Education Sciences, Ministry of Education, China: Institute for Brain Research and Rehabilitation, South China Normal University, Guangzhou, China

^4^ Philosophy and Social Science Laboratory of Reading and Development in Children and Adolescents (South China Normal University), Ministry of Education, China

**Supplemental Information**


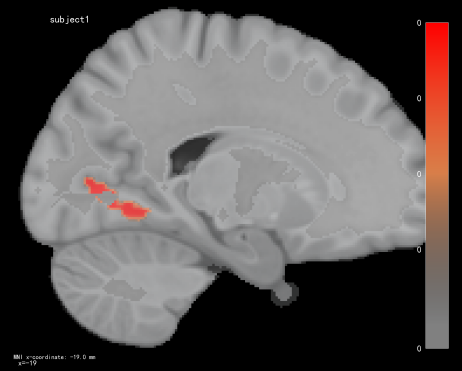

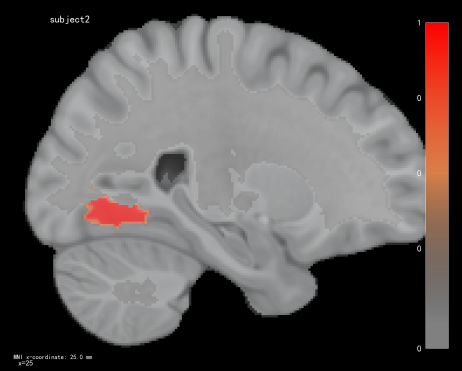

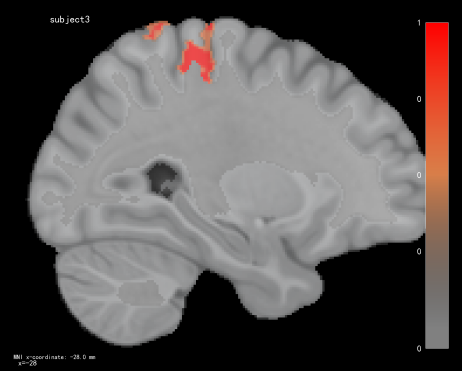

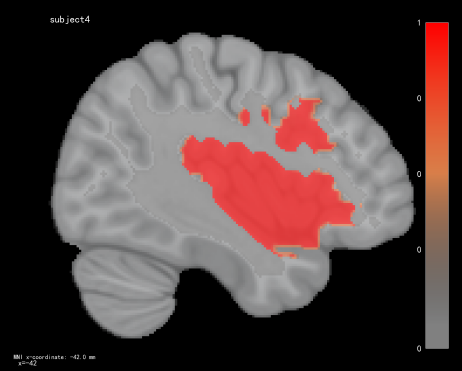

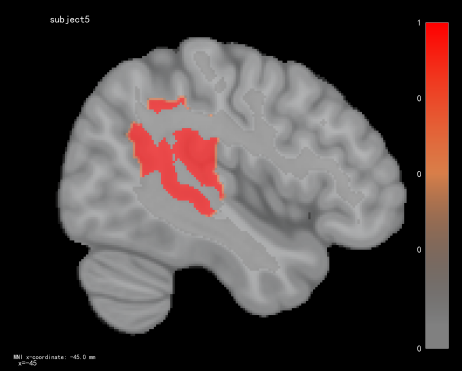

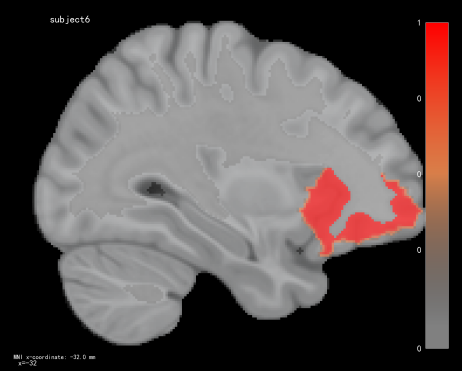

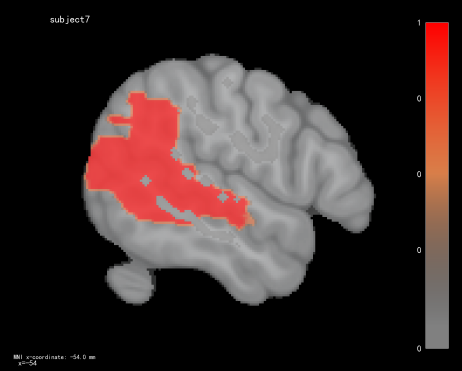

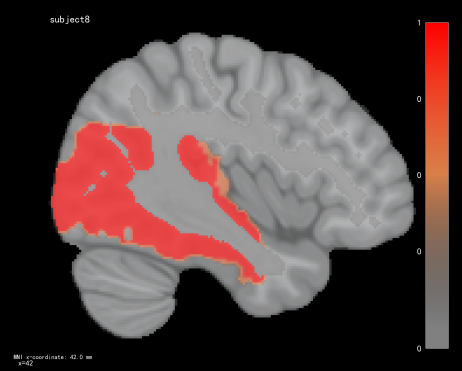

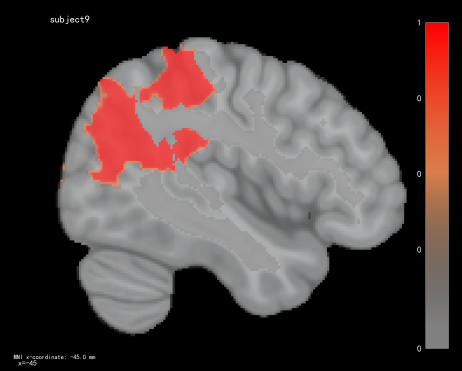

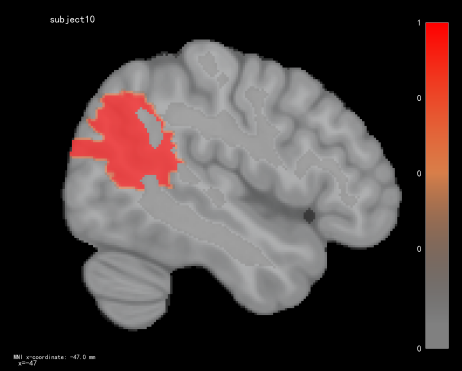

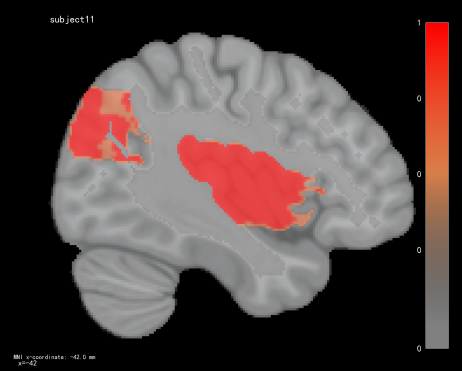

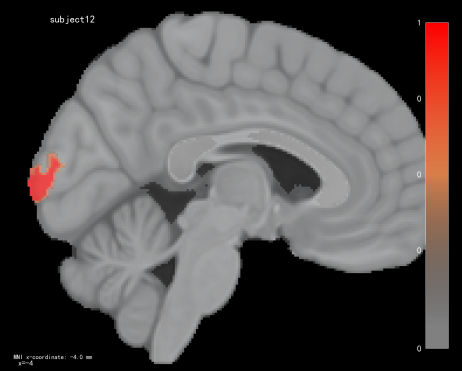

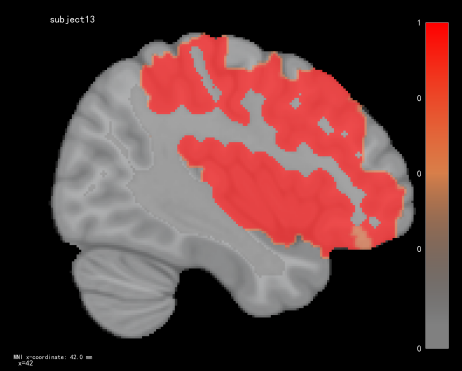

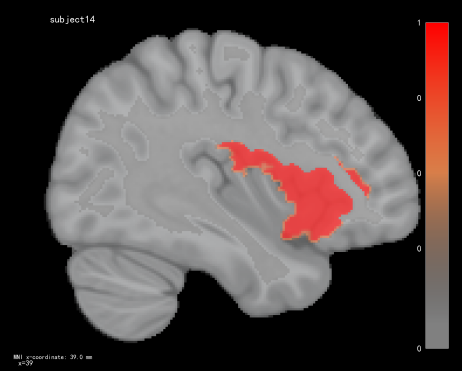

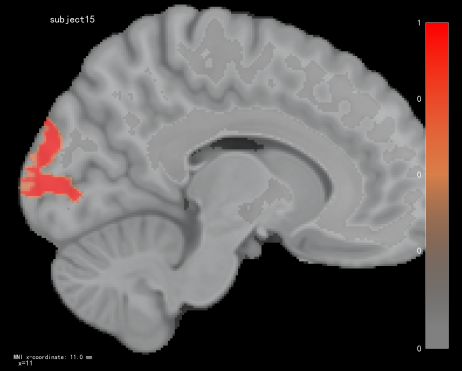


1. Stroke patients with lesions located in the cortex (n=17)


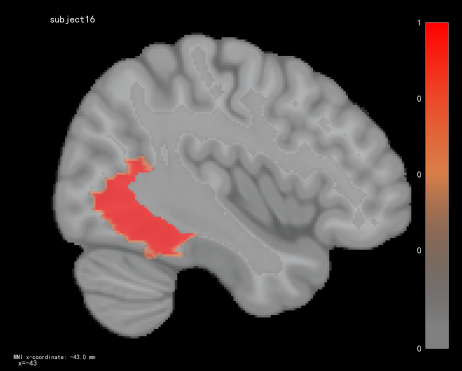

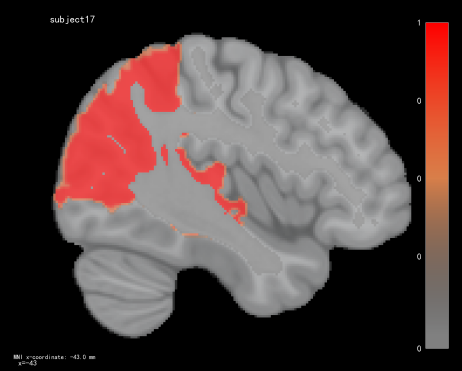

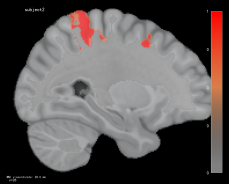

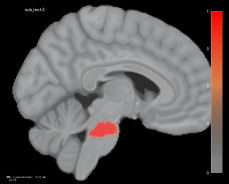

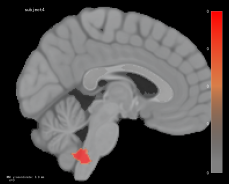

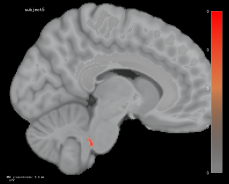

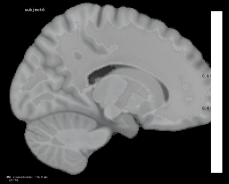

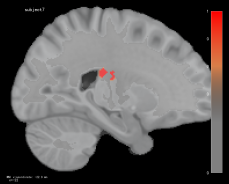

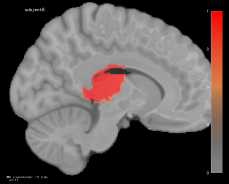

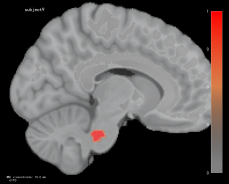

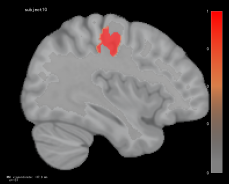

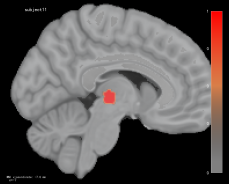

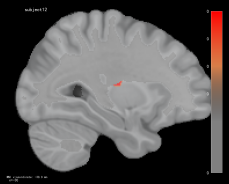

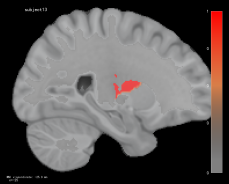

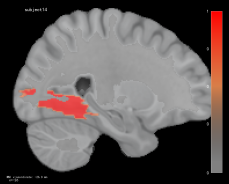

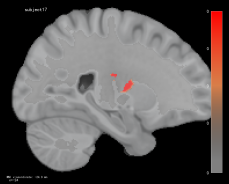

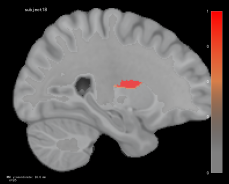

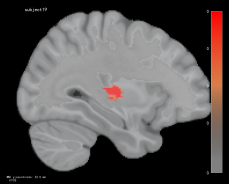

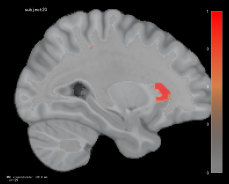

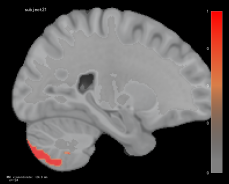

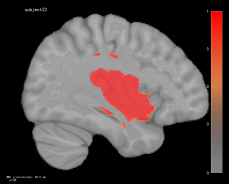

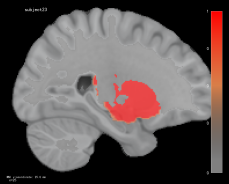

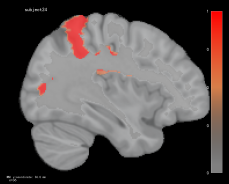

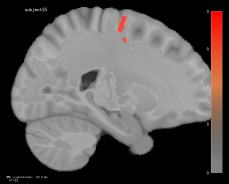

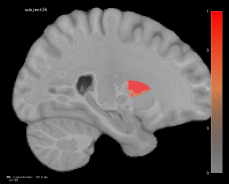

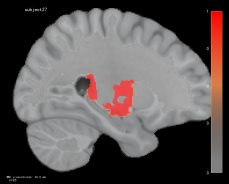

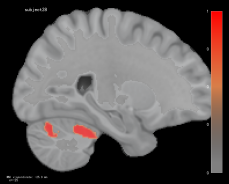

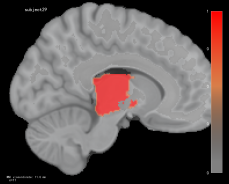

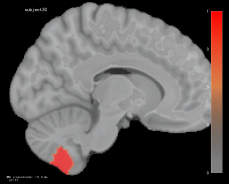

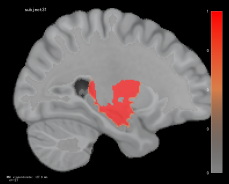

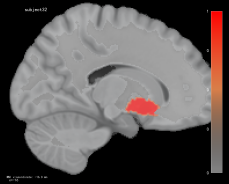

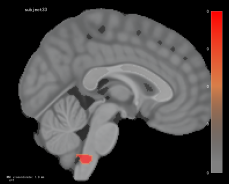

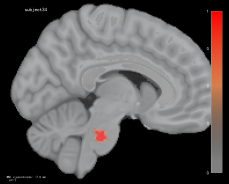

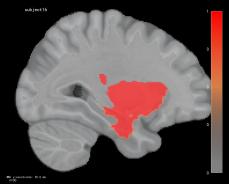


1. Stroke patients with lesions located in the subcortical regions (n=34)

Supplementary Figure 1 Categorizes stroke patients based on the anatomical location of lesions and the extent of damage (lesion volume exceeding 50%). A. Stroke patients with lesions located in the cortex (n=17). B. Stroke patients with lesions located in the subcortical regions (n=34)

| Supplementary Table 1 Spearman analysis of DTI-ALPS in the lesioned hemisphere at 3M-S and 1Y-S with behavioral modalities | | | | | | | |
| --- | --- | --- | --- | --- | --- | --- | --- |
| **Variable** | 3M-S | | | | 1Y-S | | |
|  | | **r** | **p_value** | **FDR_p** | **r** | **p_value** | **FDR_p** |
| Language | | 0.133 | 0.359 | 0.403 | 0.119 | 0.436 | 0.545 |
| MotorL | | 0.316 | **0.026^*^** | 0.163 | 0.217 | 0.162 | 0.488 |
| MotorR | | 0.300 | **0.034^*^** | 0.163 | 0.150 | 0.338 | 0.488 |
| AttentionVF | | 0.152 | 0.293 | 0.403 | 0.107 | 0.501 | 0.556 |
| AttentionAve | | -0.131 | 0.363 | 0.403 | 0.153 | 0.333 | 0.488 |
| AttentionValDis | | 0.092 | 0.526 | 0.526 | -0.186 | 0.238 | 0.488 |
| MemoryS | | 0.280 | **0.049^*^** | 0.163 | -0.066 | 0.670 | 0.670 |
| MemoryV | | 0.240 | 0.093 | 0.176 | 0.221 | 0.149 | 0.488 |
| NIHSS | | -0.229 | 0.106 | 0.176 | -0.136 | 0.341 | 0.488 |
| Infarct_volume | | -0.231 | 0.103 | 0.176 | -0.195 | 0.170 | 0.488 |
| *Unadjusted p < 0.05 | | | | | | | |
| Supplementary Table 2 Spearman analysis of 3M-S cortical and subcortical lesions and DTI-ALPS | | | | | | | |
| **Variable** | | Cor-lesion | | | Sub-lesion | | |
|  |  | **r** | **p_value** | **FDR_p** | **r** | **p_value** | **FDR_p** |
| Language | | -0.025 | 0.926 | 0.926 | 0.303 | 0.086 | 0.747 |
| MotorL | | 0.392 | 0.119 | 0.395 | 0.160 | 0.373 | 0.747 |
| MotorR | | 0.422 | 0.092 | 0.395 | 0.000 | 0.999 | 0.999 |
| AttentionVF | | 0.316 | 0.217 | 0.399 | 0.128 | 0.479 | 0.798 |
| AttentionAve | | -0.185 | 0.477 | 0.597 | -0.085 | 0.637 | 0.866 |
| AttentionValDis | | -0.136 | 0.604 | 0.671 | -0.002 | 0.993 | 0.999 |
| MemoryS | | 0.241 | 0.352 | 0.503 | 0.183 | 0.309 | 0.747 |
| MemoryV | | 0.482 | 0.050 | 0.395 | 0.219 | 0.222 | 0.747 |
| NIHSS | | -0.358 | 0.158 | 0.395 | -0.070 | 0.693 | 0.866 |
| Infarct_volume | | -0.301 | 0.240 | 0.399 | -0.160 | 0.367 | 0.747 |
|  | |  |  |  |  |  |  |
| Supplementary Table 3 Spearman analysis of 1Y-S cortical and subcortical lesions and DTI-ALPS | | | | | | | |
| **Variable** | | Cor-lesion | | | Sub-lesion | | |
|  |  | **r** | **p_value** | **FDR_p** | **r** | **p_value** | **FDR_p** |
| Language | | 0.018 | 0.950 | 0.992 | 0.099 | 0.601 | 0.751 |
| MotorL | | 0.134 | 0.648 | 0.992 | 0.000 | 0.998 | 0.998 |
| MotorR | | 0.046 | 0.876 | 0.992 | 0.129 | 0.506 | 0.751 |
| AttentionVF | | 0.257 | 0.375 | 0.856 | 0.027 | 0.890 | 0.989 |
| AttentionAve | | 0.626 | **0.017^*^** | 0.165 | 0.125 | 0.527 | 0.751 |
| AttentionValDis | | 0.090 | 0.759 | 0.992 | -0.150 | 0.446 | 0.751 |
| MemoryS | | -0.284 | 0.326 | 0.856 | -0.111 | 0.561 | 0.751 |
| MemoryV | | 0.305 | 0.288 | 0.856 | 0.239 | 0.203 | 0.751 |
| NIHSS | | 0.003 | 0.992 | 0.992 | -0.095 | 0.592 | 0.751 |
| Infarct_volume | | -0.206 | 0.428 | 0.856 | -0.110 | 0.537 | 0.751 |
| *Unadjusted p < 0.05 | | | | | | | |

**Abbreviations**

1. PSCI: Post-stroke cognitive impairment
2. GS: glymphatic system
3. DTI-ALPS: diffusion tensor imaging analysis along the perivascular space
4. Aβ: β-amyloid
5. CSF: cerebrospinal fluid
6. ISF: interstitial fluid
7. PVS: perivascular space
8. PCA: principal component analysis
9. FA: fractional anisotropy
10. MD: mean diffusivity
11. AD: axial diffusivity ,
12. RD: radial diffusivity
13. AQP4: aquaporin-4
14. ICC: intraclass correlation coefficients
15. sICH: spontaneous intracerebral hemorrhage
