# Supplementary material for: Chronic Glymphatic Dysfunction Modulates Domain‐Specific Cognitive Recovery After Stroke: A DTI‐ALPS Lesion Stratification Study
Source: CNS Neurosci Ther. 2025 Jul 14;31(7):e70512. doi: 10.1111/cns.70512 (PMC12256992; doi:10.1111/cns.70512)
Supplement: Supplementary file 2 — Data S2. [file CNS-31-e70512-s001.pdf]

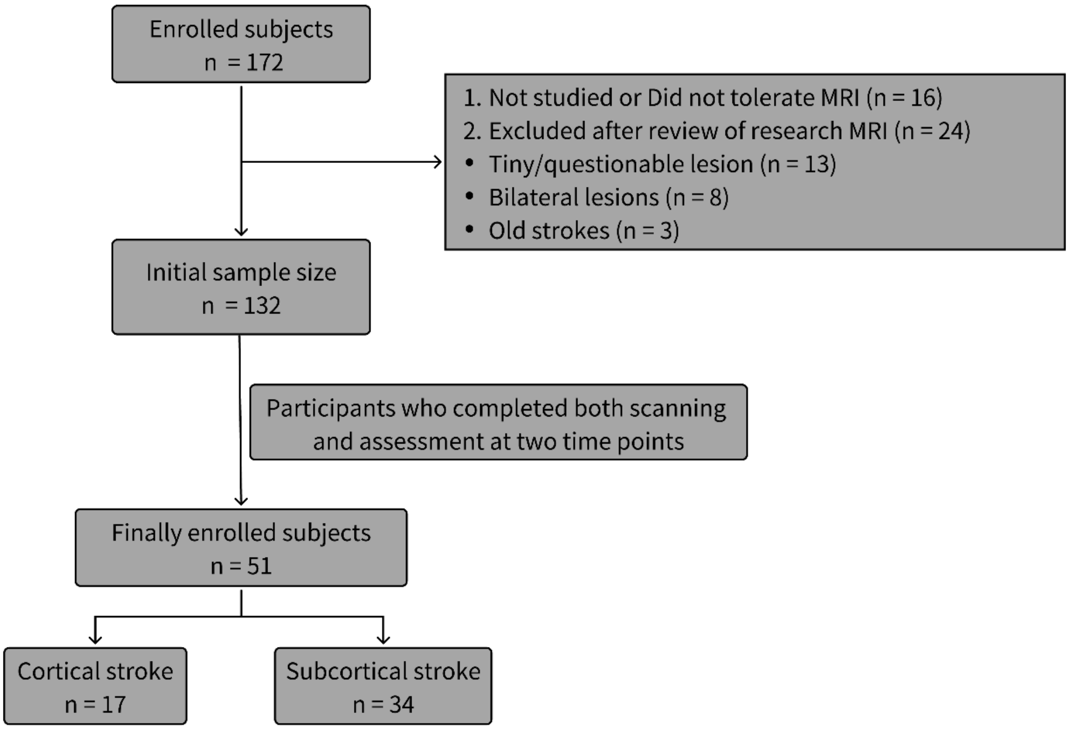

Full unedited gel/blot for Figure 1

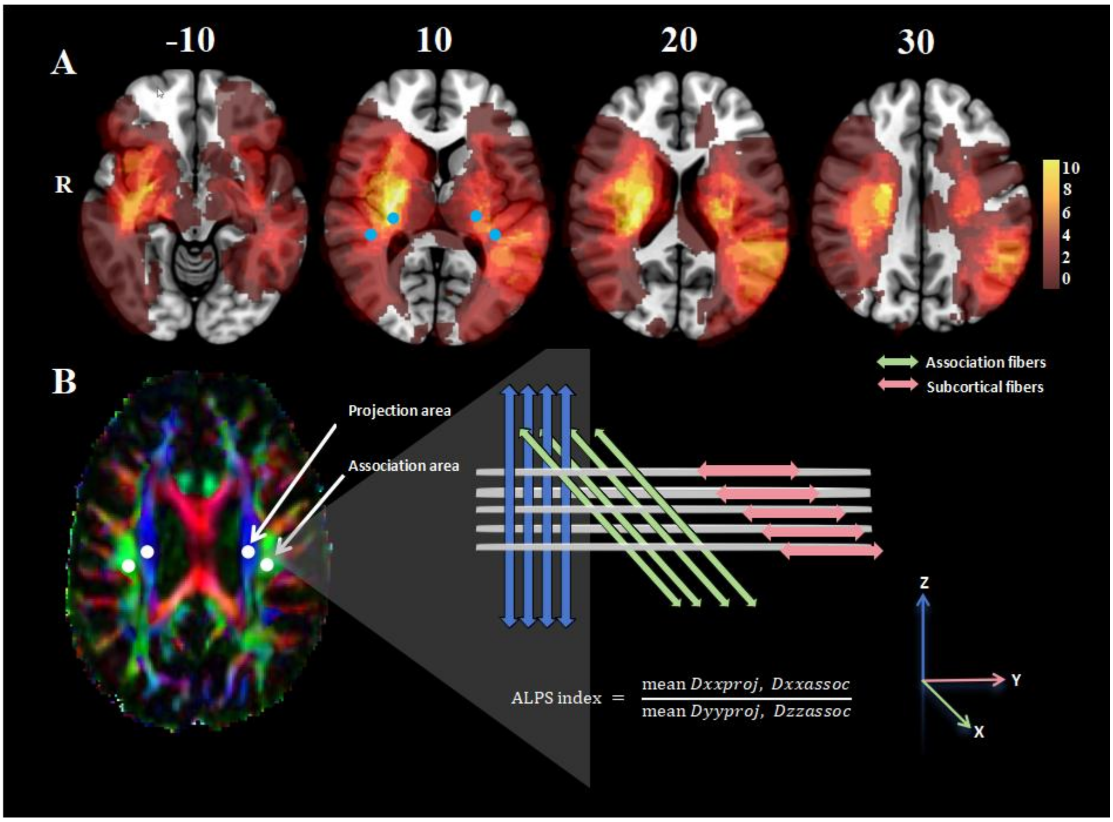

Full unedited gel/blot for Figure 2

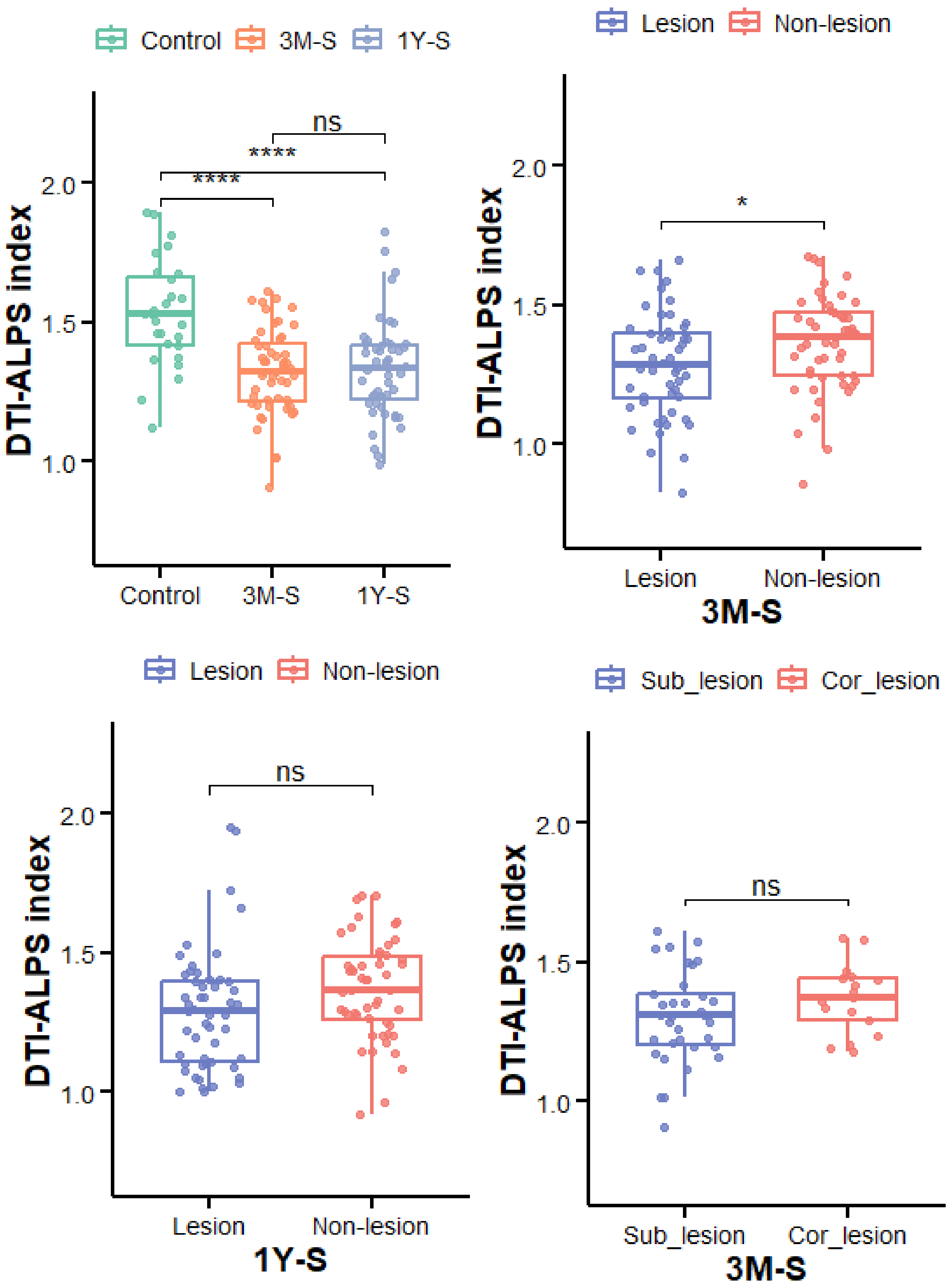

Control
3M-S
1Y-S
ns
****
****
DTI-ALPS index
2.0
1.5
1.0
Control
3M-S
1Y-S
Lesion
Non-lesion
*
DTI-ALPS index
2.0
1.5
1.0
Lesion
Non-lesion
3M-S
Lesion
Non-lesion
ns
DTI-ALPS index
2.0
1.5
1.0
Lesion
Non-lesion
1Y-S
Sub_lesion
Cor_lesion
ns
DTI-ALPS index
2.0
1.5
1.0
Sub_lesion
Cor_lesion
3M-S

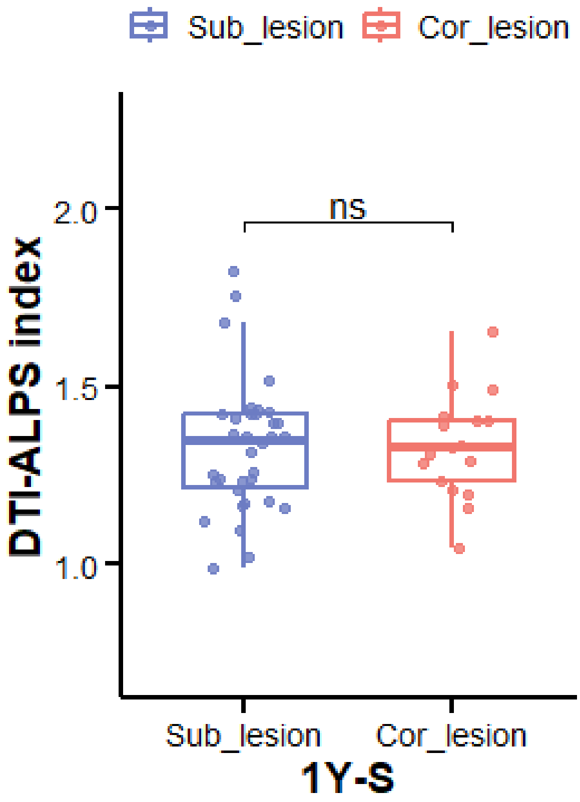

Full unedited gel/blot for Figure 3
